# Pharmacokinetic and Pharmacodynamic integration of tilmicosin against *Mycoplasma gallisepticum* in the target infection site in chickens

**DOI:** 10.3389/fvets.2022.952599

**Published:** 2022-09-29

**Authors:** Nan Zhang, Minghu Zhou, Xiu Yan, Jinxin Liu, Sheng Yuan, Hong Yang, Huanzhong Ding, Dexian Zhang, Yinshan Bai

**Affiliations:** ^1^School of Life Science and Engineering, Foshan University, Foshan, China; ^2^Guangdong Key Laboratory for Veterinary Drug Development and Safety Evaluation, South China Agricultural University, Guangzhou, China

**Keywords:** Pharmacokinetic, Pharmacodynamic, tilmicosin, *Mycoplasma gallisepticum*, the target infection site

## Abstract

*Mycoplasma gallisepticum* (*M. gallisepticum*) is a primary respiratory pathogen of poultry and causes significant economic losses to the poultry industry. There were no reported articles concerning the Pharmacokinetics/Pharmacodynamics (PK/PD) interactions of tilmicosin against *M. gallisepticum in vivo*. In the current study, we established an *in vivo M. gallisepticum* infection model and tilmicosin was administered orally to the *M. gallisepticum*-infected chickens by different dosage regimens. The concentration of tilmicosin in lung tissue was determined by high-pressure liquid chromatography/tandem mass spectrometry (HPLC–MS/MS), besides the counting of the viable colony of *M. gallisepticum* in lung tissue was also monitored dynamically to appraise the PK/PD interactions of tilmicosin against *M. gallisepticum*. We found that anti-mycoplasmal activity was concentration-dependent and mycoplasmacidal activity was observed at tilmicosin dosage >7.5 mg/kg. The PK/PD parameter of AUC/MIC (The area under the concentration–time curve divided by the minimal inhibitory concentration) correlated well with anti-mycoplasmal efficacy (*R*^2^ = 0.92). The ratios of AUC/MIC for 1 log_10_ and 3 log_10_ colony-forming units [CFU]/lung reductions were 300.02 and 6,950.15 h, respectively. These findings indicated that tilmicosin may be therapeutically effective in chickens to treat *M. gallisepticum* lung infections if administered at a dose of 9.12 mg/kg.

## Introduction

*Mycoplasma gallisepticum* (*M. gallisepticum)* is the primary causative agent of chronic respiratory disease in chickens and sinusitis in turkeys. The primary symptoms of these infections are air sacculitis, nasal discharge, and keratoconjunctivitis; and *M*. *gallisepticum* can be transmitted horizontally as well as vertically through eggs (1). Embryo mortality, weight loss, and lowered egg production are adverse consequences of these infections in chickens and places an economic burden on these farms (2). Pleuromutilins, tetracyclines, quinolones, and macrolides are the current modalities used forinfection control on poultry farms for *M. gallisepticum* infections (3, 4). The Pharmacokinetics (PK) of macrolides are distinctive due to their large distribution volume and persistence and retention in the lung, and are the preferred treatment for bacterial respiratory diseases of livestock (5). However, macrolide resistance in *M. gallisepticum* has appeared and a more reasonable dosing schedule should be established using PK/Pharmacodynamics (PD) modeling to ensure proper treatments are established (6, 7).

Tilmicosin is a semi-synthetic 16-membered lactone macrolide with robust antimicrobial activity *in vitro* against Gram-positive and Gram-negative bacteria, as well as *Mycoplasma* spp. (8). It is approved to treat respiratory disease in cattle and sheep caused by *Mannheimia haemolytica*, in swine caused by *Actinobacillus pleuropneumoniae* and *Pasteurella multocida* and in chickens and swine caused by *M. gallisepticum* and *Mycoplasma hyopneumoniae* (9). Tilmicosin is also widely used to treat the respiratory disease caused by *M. gallisepticum* due to its accumulation in /^*^98lung and high levels are retained in lung tissue. This is a desirable effect since *M. gallisepticum* is a facultative intracellular pathogen and lung tissue levels should be used when developing treatment protocols for poultry (10, 11). In addition, PK/PD integration models of tilmicosin against *M. gallisepticum and M. hyopneumoniae* have only been performed using *in vitro* models and studies using mycoplasma-infected chickens are lacking (12, 13).

The purpose of the current study was to establish an *in vivo* PK/PD model based on different dosing regimens for tilmicosin in chicken lung tissues and evaluate its antibacterial activity against *M. gallisepticum*. This study provides a baseline reference for optimizing tilmicosin treatments for mycoplasma infections in chickens.

## Materials and methods

### Organisms and chemicals

*Mycoplasma gallisepticum* strain S6 was obtained from the Chinese Veterinary Microorganism Culture Collection Center (Beijing, China). Culture media consisted of *M. gallisepticum* artificial medium base (Qingdao Hope Biological Technology) containing nicotinamide adenine dinucleotide and cysteine and swine serum (Guangzhou Ruite Biological) as previously described (3). Tilmicosin phosphate was a kind gift of Eastern Along Pharmaceuticals (Guangdong, China). Acetonitrile, formic acid, methanol (high-performance liquid chromatography grade), and remaining analytical grade reagents were purchased from Guangzhou Chemical Reagent.

### Animals and inoculations

Specific pathogen-free (SPF) 3-day-old White Leghorn chickens were purchased from Guangdong Da Hua Nong Animal Health (Guangdong, China). All *in vivo* experiments were approved by the animal research committees of Foshan University Animal Ethics Committee (Approval number: 2019044). The chickens were given antimicrobial-free feed and water *ad libitum*. Chickens were infected with *M. gallisepticum* as previously described (14). The 50 ml logarithmic growth phase of *M. gallisepticum* culture was centrifuged 20 min at 8,000r, then the supernatant was discarded and the residue was dissolved in 2 ml of *M. gallisepticum* culture. 0.2 ml aliquots of 10^9^ colony-forming units [CFU]/ml were administered twice a day *via* intratracheal injection for three consecutive days. The animals were continually monitored for clinical signs of respiratory infection, such as nasal discharge, sneezing, coughing, and rales. The successful infection model was confirmed by the clinical signs as well as re-isolation and identification of the pathogen and the presence of anti-*M. gallisepticum* antibodies using a commercial ELISA kit (Shenzhen Lvshiyuan Biotechnology Co., Ltd).

### Determination of the minimal inhibitory concentration

The minimal inhibitory concentration (MIC) of tilmicosin against strain S6 was determined using a modified MIC assay method (15). Briefly, log phase cultures were diluted with medium to 10^5^ CFU/ml and added to a 96-well plate that contained two-fold serial dilutions of tilmicosin from a 0.25 μg/ml stock. Control wells included a growth control (lacking tilmicosin), an end-point control (blank medium at pH 6.8), and a sterility control (sterile medium at pH 7.8). The plates were incubated at 37°C in a 5% CO_2_ humidified atmosphere until the growth and end-point controls were of the same color. The minimal drug concentration that caused no color change was defined as the MIC.

### PK of tilmicosin in *M. gallisepticum*-infected chickens

We infected 240 chickens and tilmicosin was orally administered at 1 and 30 mg/kg (equal numbers) and 8 chickens per group were euthanized at 0.083, 0.25, 0.5, 1, 2, 4, 6, 8, 12, 24, 48, 72, 96, 120, and 144 h following drug administration and lung tissues were collected. A control group of eight infected but untreated chickens were euthanized at the start of the experiments. Lung tissues were stored at −20°C and analyzed within 2 weeks.

Tilmicosin concentrations in lung tissues were determined by high-pressure liquid chromatography/tandem mass spectrometry (HPLC–MS/MS) using a Shimadzu LCMS-8045 triple quadrupole a triple quadrupole mass spectrometer. Chromatographic separations were performed using a Shim-pack GIST-HP C18 (50 mm × 21 mm; 3 μm) column using a mobile phase of solution A (0.1% formic acid in water) and solution B (acetonitrile) at a flow rate of 0.3 ml/min. The gradient elution was 0–1.5 min, 15% B; 1.5–4 min, 65% B; 4–4.5 min, 95% B; 4.5–5.5 min, 95% B; and 5.5–10 min, 15% B. The injection volume was 5 μl. Lung tissues were treated as previously described with modifications (16). In brief, lung tissue samples were homogenized and acetonitrile was added to extract tilmicosin. The mixture was vortexed and centrifuged, then 9 ml of water was added into the supernatants and purified using an SPE C18 cartridge and tilmicosin was eluted using 3 ml acetonitrile. The eluate was evaporated to dryness under a gentle stream of nitrogen at 45°C and the residue was dissolved in 1 ml of mobile phase solvent and filtered through a 0.22 mm syringe filter prior to HPLC–MS/MS analysis.

The PK profiles of tilmicosin in lung tissues were analyzed using a noncompartmental model provided in WinNonlin software, Version 6.1 (Pharsight, Mountain View, CA, USA). PK parameters include elimination half-life (*t*_1/2_), the area under the concentration–time curve (AUC), and maximum concentration of drug in samples (*C*_max_), the time of peak concentration (*t*_max_), and mean residence time (MRT).

### Pharmacodynamics of tilmicosin in *M. gallisepticum*-infected chickens

A group of 168 *M. gallisepticum*-infected chickens (see above) were administered tilmicosin by oral gavage at 1, 2, 4, 7.5, 10, 15, and 30 mg/kg. A control group of 24 infected chickens was administrated the same volume of normal saline. Six chickens at each sampling time point per group were euthanized at 0, 24, 48, and 72 h and the lung tissues were aseptically collected and homogenized in 1 ml of culture medium and spread-plated onto agar plates containing culture medium. A reduction in CFU <3 log_10_ was denoted a bacteriostatic and ≥3 log_10_ CFU indicated a bactericidal effect.

### Pharmacokinetics/Pharmacodynamics analysis

The PK/PD index of AUC/MIC (The area under the concentration–time curve divided by the MIC) was calculated using MIC value and PK parameter derived from tilmicosin levels in lung tissue samples. The PK of tilmicosin in *M. gallisepticum-*infected chickens at the dose of 4, 7.5, and 10 mg/kg was established using our preliminary results (16). Drug effectiveness was reflected as a reduction in viable counts from lung tissues after each treatment compared to the control group. The PK/PD was fitted by linear model, E_max_ model, and Sigmoid E_max_ model, respectively. The following equation was exhibited as the linear model:


(1) $$\begin{array}{l} Y=AX+B \end{array}$$

Where Y is the antibacterial effect that was assessed as the reduction in log_10_ CFU/lung after each administration of tilmicosin, compared to the log_10_ CFU/lung in the untreated control group (absence of tilmicosin); X is the AUC/MIC after the different administrations. A is the slope and B is the intercept.

The following equation was response for the E_max_ model:


(2) $$\begin{array}{l} E\;=\;E_{0}\;+\;\frac{\left(E_{\max}-E_{0}\right)\times C}{EC_{50}+C} \end{array}$$

Where E is the antibacterial effect that was assessed as the reduction in log_10_ CFU/lung after each administration of tilmicosin, compared to the log_10_ CFU/lung in the untreated control group (absence of tilmicosin); E_max_ is the difference in effect between the greatest amount of growth (as seen for the growth control, E_0_) and the greatest amount of kill; C is the AUC/MIC after the different administrations; EC_50_ is the PK/PD parameter producing a 50% reduction in *M. gallisepticum* counts.

The following equation was response for the sigmoid E_max_ model:


(3) $$\begin{array}{l} E\;=\;E_{0}\;+\;\frac{E_{\max}\times C_{e}^{N}}{EC_{50}^{N}+C_{e}^{N}} \end{array}$$

Where E is the antibacterial effect that was assessed as the reduction in log_10_ CFU/lung after each administration of tilmicosin, compared to the log_10_ CFU/lung in the untreated control group (absence of tilmicosin); E_max_ is the difference in effect between the greatest amount of growth (as seen for the growth control, E_0_) and the greatest amount of kill; C_e_ is the PK/PD parameter in the effect compartment; EC_50_ is the PK/PD parameter producing a 50% reduction in *M. gallisepticum* counts, and N is the Hill coefficient that describes the steepness of the effect curve of AUC/MIC.

### Dosage calculation

An optimal regimen was established using the dose required for different magnitudes of efficiency provided by the following equation as previously described (17).


(4) $$\begin{array}{l} \text{Dose}\left(\text{per day}\right)=\frac{{\left(\text{AUC}/\text{MIC}\right)}_{\text{breakpoint}}\times \text{MIC}\times \text{CL}}{\text{F}} \end{array}$$

Where dose (per day) is the optimal dose (mg/kg.bw); AUC/MIC is the targeted endpoint for the desired effect (Lh/kg); MIC_90_ is the 90% of the MIC distribution (mg/L); and clearance is the lung clearance expressed as kg/kg/h (CL per h was 0.373 kg/kg/h). F is the bioavailability.

## Results

### *Mycoplasma gallisepticum* infection model

**Figure 1:**
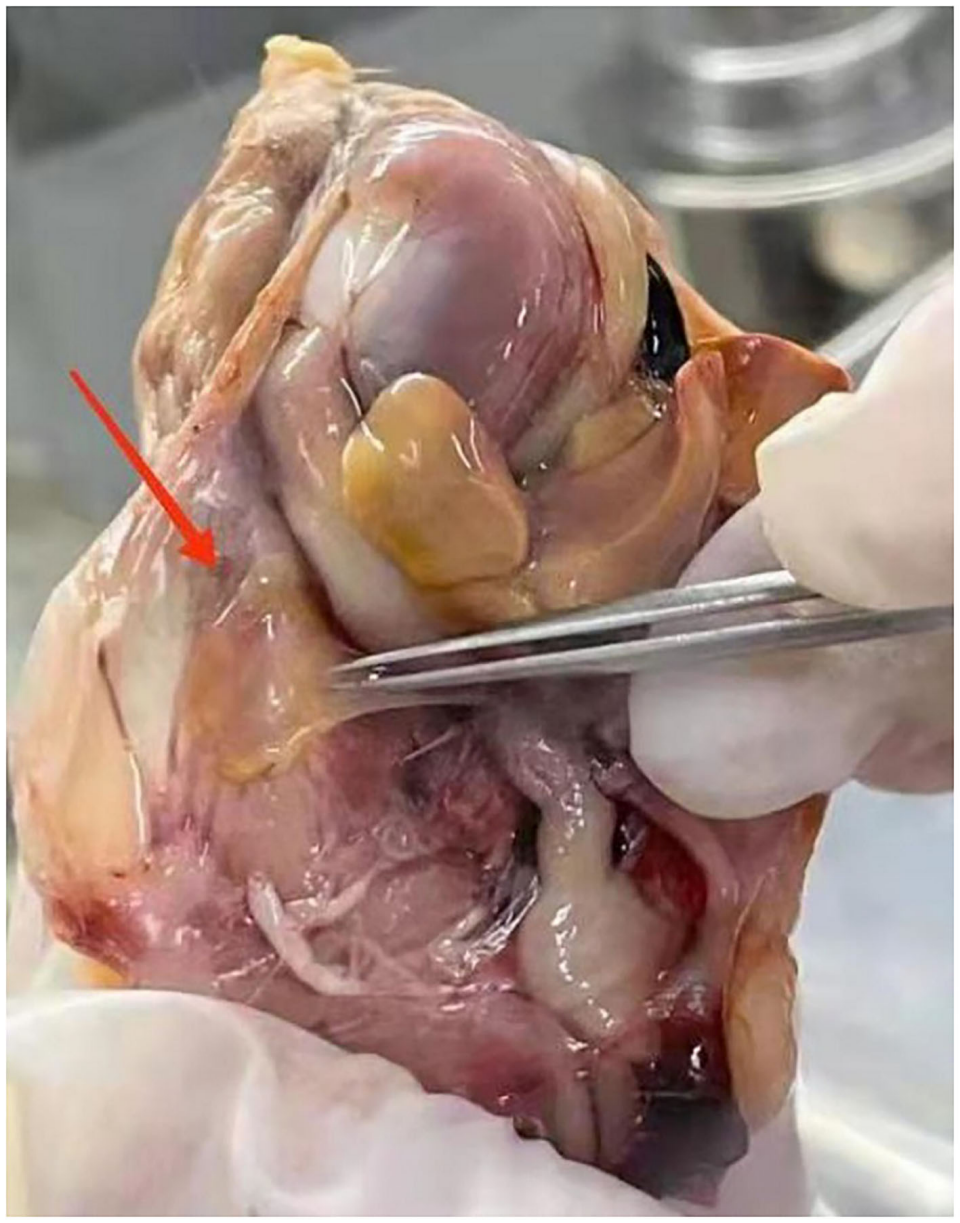
The symptoms of air sac in *Mycoplasma gallisepticum* (*M. gallisepticum*)-infected chickens.

*Mycoplasma gallisepticum-*infected chickens in our experiments were identified based on observable clinical signs, such as coughs, sneezing, ocular, and nasal discharge, breathing difficulty, and moist rales. Air sacs were thickened and contained opaque and caseous deposits (Figure 1). These infections were confirmed in subsequent ELISA tests that were positive for *M. gallisepticum* antibody and the CFU of *M gallisepticum* that produced characteristic nipple-shaped or fried egg colony shapes on solid media.

### PK analysis

A tilmicosin calibration curve was used to quantify drug levels in lung tissue and was linear from 0.002 to 0.5 μg/ml. The limits of detection (LOD) and quantification (LOQ) were 0.004 and 0.008 μg/g, respectively, and recoveries from lung tissue spiked samples ranged from 80.53 to 86.53% in lung tissue homogenates. The within-run and between-run relative standard deviations were 3.64 and 8.92%, respectively.

**Figure 2:**
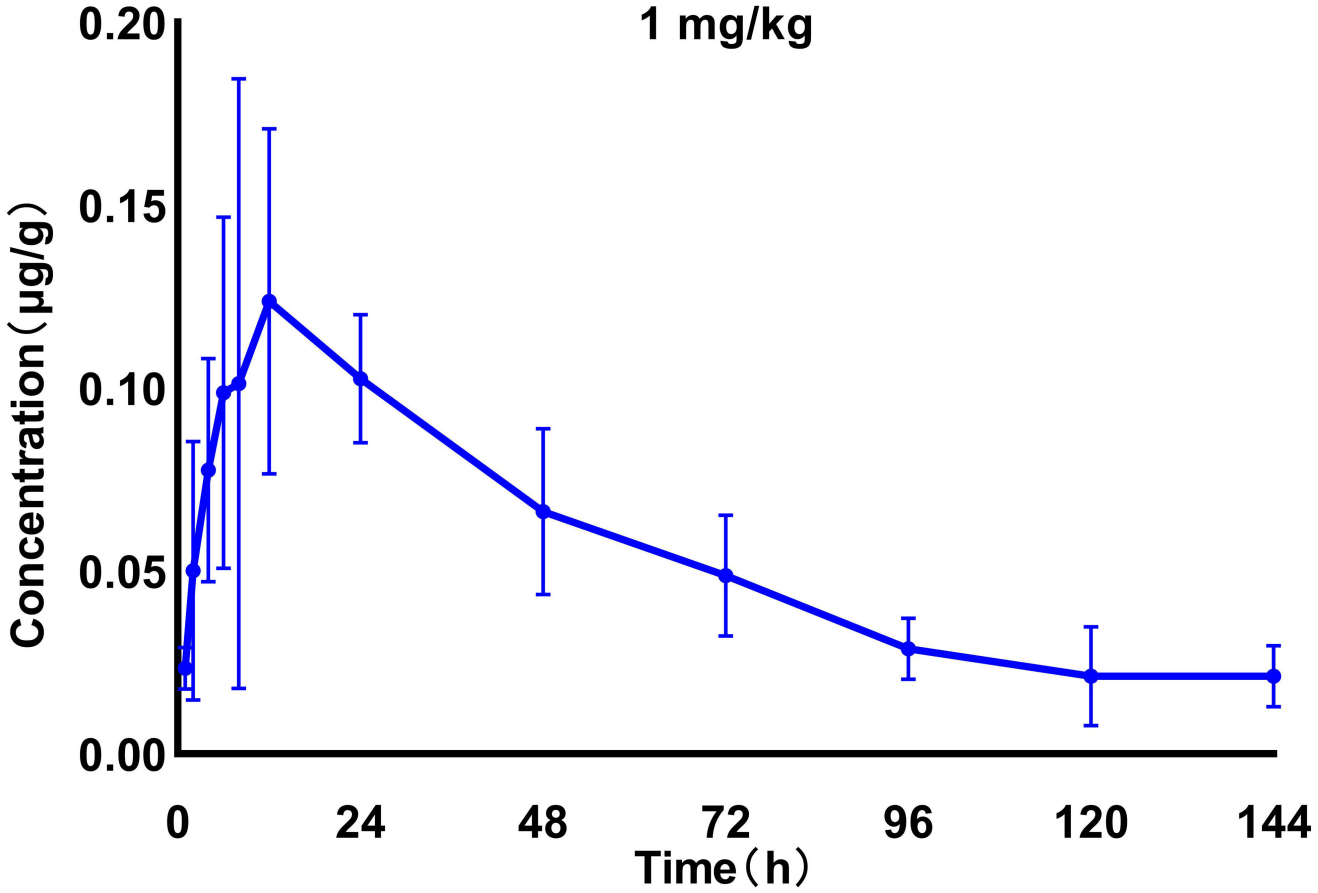
Time–concentration curves of tilmicosin in lung tissues after oral administration of 1 mg/kg in infected *M. gallisepticum* chickens (*n* = 8/time point).

**Figure 3:**
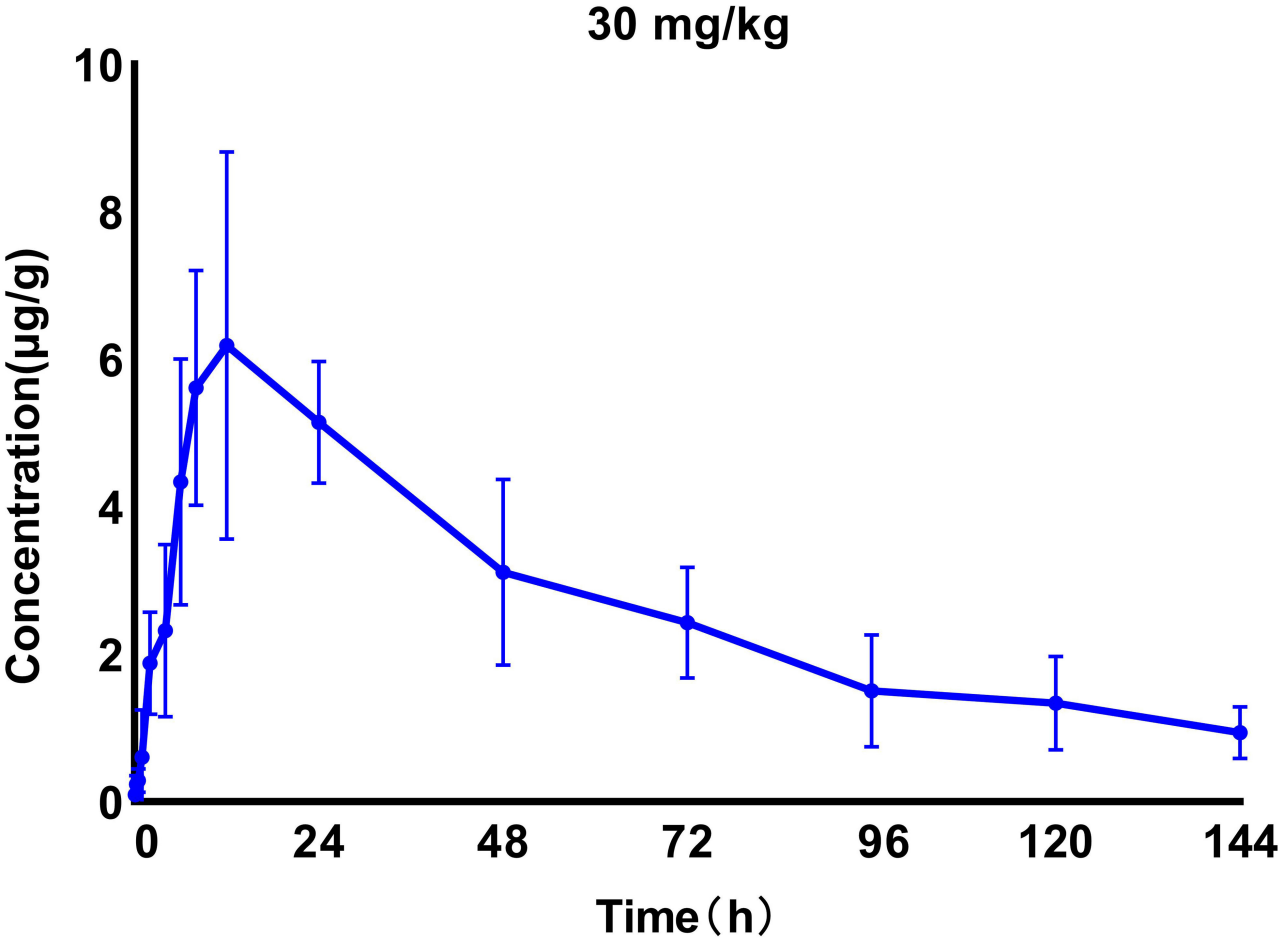
Time–concentration curves of tilmicosin in lung tissues after oral administration of 30 mg/kg in *M. gallisepticum*-infected chickens (*n* = 8/time point).

We generated time–concentration curves of tilmicosin in lung tissues after oral administration of 1 and 30 mg/kg in our infected animals. There were no differences in *t*_1/2_, *t*_max_ MRT, and CL between the two doses (*p* > 0.5) 0.1, 4, 7.5, 10, and 30 mg/kg doses generated a significant correlation (*R*^2^ > 0.97) between dose and AUC (Figures 2, 3; Table 1).

**Table 1:** The main PK parameters of tilmicosin in lung tissues of the *M. gallisepticum* infected chickens.

**Parameter**	**Units**	**1 mg/kg**	**30 mg/kg**
t_1/2kel_	h	48.46	50.60
T_max_	h	12.00	12.00
C_max_	μg/g	0.12	6.19
AUC	μg·h/g	13.62	464.23
MRT	h	53.60	50.88
CL/F	l/h/kg	0.09	0.06

t_1/2*kel*_, elimination half-life; t_*max*_, the time of peak concentration; C_*max*_, the maximum concentration of drug in samples; AUC, the area under the concentration–time curve; MRT, mean residence time; CL/F, clearance divided by bioavailability.

### Pharmacodynamics of tilmicosin against *M. gallisepticum* in infected chickens

**Figure 4:**
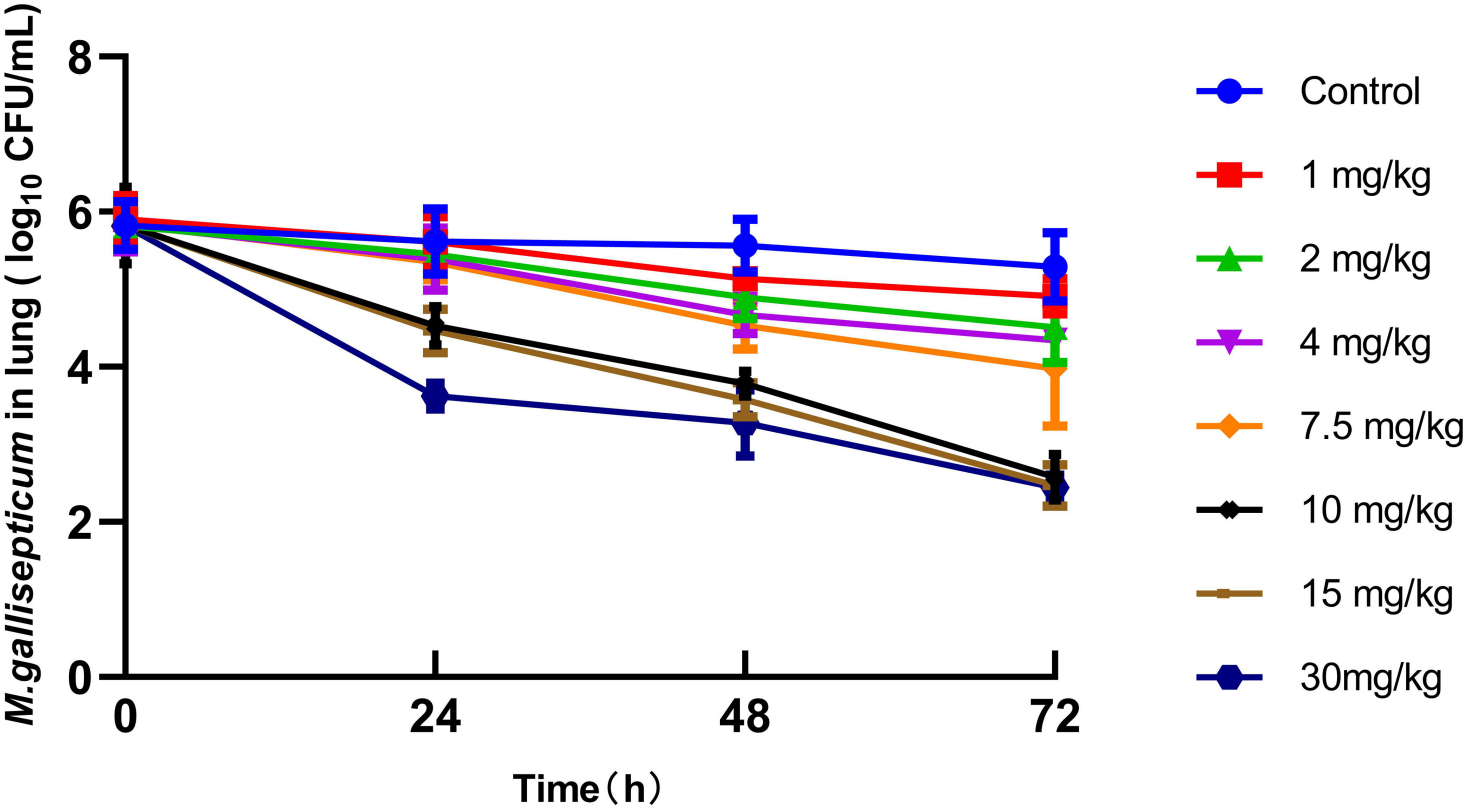
Viable counts (colony-forming units [CFU]) of *M. gallisepticum* in lung tissues after different regimens of tilmicosin (*n* = 6/time point).

The PD parameters using different regimens indicated that the tilmicosin concentration was directly proportional to anti-mycoplasmal activity at doses >7.5 mg/kg. However, there were no differences in the reduction of the CFU of *M gallisepticum* between 10, 15, and 30 mg/kg at 72 h post-administration (Figure 4).

### PK/PD analysis

**Figure 5:**
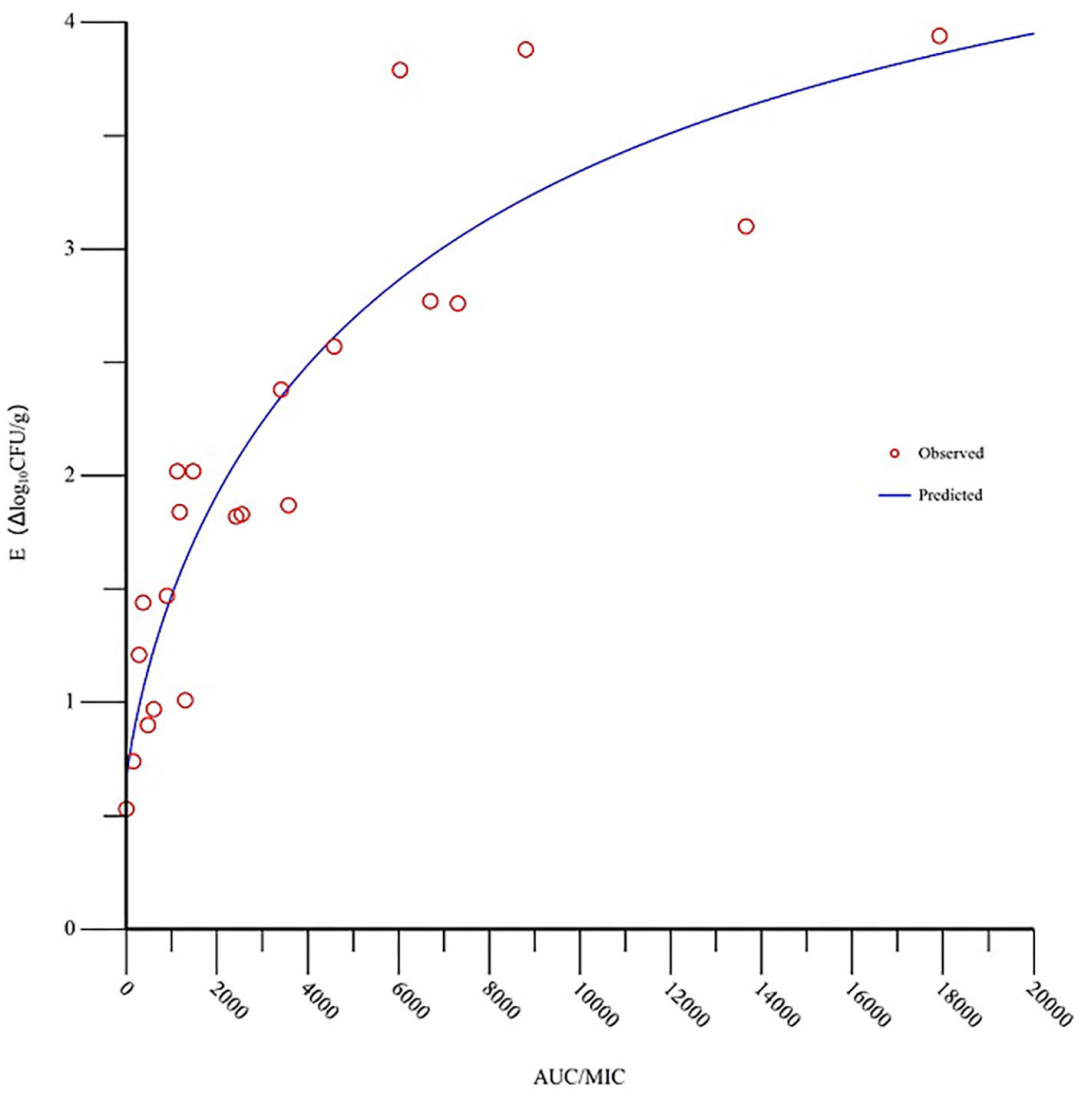
Sigmoid *E*_max_ relationships for the anti-mycoplasmal effect (E, log_10_ CFU/lung) and the *in vivo* AUC/MIC (the area under the concentration-time curve divided by the minimal inhibitory concentration) ratio against *M. gallisepticum* in the lung tissues of chickens.

The MIC of tilmicosin against *M. gallisepticum* was 0.0156 μg/ml. The AUC/MIC and the reduction in viable counts in different administration regimens are displayed in Table 2. The fitness of the PK/PD employed by the linear model, E_max_ model, and Sigmoid E_max_ model were 0.75, 0.90, and 0.92, respectively. Therefore, the Sigmoid E_max_ model was selected to calculate the PK/PD parameters, which were shown in Table 3. The PK/PD parameter vs. antimycoplasmal effect curve was displayed in Figure 5. The dose–response relationships analyzed using the Sigmoid E_max_ model indicated AUC/MIC for 1 log_10_ and 3 log_10_ CFU/lung reductions were 300.02 and 6,950.15 h, respectively.

**Table 2:** The Pharmacokinetics/Pharmacodynamics (PK/PD) parameter of AUC/MIC (the area under the concentration–time curve divided by the minimal inhibitory concentration) and the corresponding antimycoplasmal effect in various administration regimens of tilmicosin.

**Dose (mg/kg)**	**Time (h)**	**E (ΔLog_10_CUF/g)**	**AUC/MIC (h)**
1	0–24	0.74	150.81
	0–48	1.21	279.78
	0–72	1.44	367.62
2	0–24	0.90	476.49
	0–48	1.47	893.51
	0–72	1.84	1,173.78
4	0–24	0.97	603.54
	0–48	1.69	1,121.12
	0–72	2.02	1,472.38
7	0–24	1.01	1,298.28
	0–48	1.83	2,544.98
	0–72	2.38	3,410.21
10	0–24	1.32	2,418.24
	0–48	2.33	4,581.45
	0–72	3.19	6,033.34
15	0–24 h	1.87	3,573.65
	0–48	2.47	6,701.35
	0–72	3.68	8,803.37
30	0–24	2.06	7,307.02
	0–48	3.10	13,660.61
	0–72 h	3.94	17,923.84

E is the antibacterial effect that was assessed as the reduction in log_10_ CFU/lung after each administration of tilmicosin, compared to the log_10_ CFU/lung in untreated control group (absence of tilmicosin).

**Table 3:** The PK/PD parameters of tilmicosin against *M. gallisepticum in vivo* using the *Emax* model employed by WinNonlin software.

**Parameter**	**Value**
E_max_	4.78
EC_50_	7,174.57
E_0_	0.64
N	0.79
R^2^	0.92

E_*max*_, the reduction in log CFU/lung for the untreated control chicken. E_0_, the maximum reduction after administration that represents the maximum antibacterial effect. EC_50_, the AUC/MIC value required to achieve 50% of the maximal antibacterial effect. N, the Hill coefficient which describes the steepness of the AUC/MIC and effect curve.

###  Dosage calculation

Since there was not enough tilmicosin MIC data available to provide an estimate of the MIC_90_, the MIC of tilmicosin against strain S6 was substituted as a projected MIC_90_. We accounted for bioavailability due to the extravascular administration route. A dosage of 9.12 mg/kg was calculated to be capable of a reduction of 3 log_10_ CFU/lung.

## Discussion

Tilmicosin has good tissue penetration and is used for the treatment of respiratory disease caused by *M. gallisepticum*. Previous reports had indicated that higher concentrations of the drug were present in lung tissues relative to plasma. For example, the PK of tilmicosin in calves revealed through bronchial swabs and bronchoalveolar lavage samples exceeded plasma levels by 5.67 and 7.76 folds, respectively (18). The PKs and tissue levels of tilmicosin in chickens demonstrate that the concentration in the lungs was greater than that of plasma (19). In our earlier study, we found that the C_max_ of tilmicosin in lung tissues was 12.8 and 14.28 times higher than for plasma between *M. gallisepticum*-infected chickens and their healthy counterparts (16). Moreover, tilmicosin has been widely used in the treatment of CRD caused by *M. gallisepticum* which mainly colonized in lung tissue. Consequently, the concentration of tilmicosin in lung tissue was more suitable for the PK/PD relationship of tilmicosin against *M. gallisepticum* in the *in vivo* infection model.

*In vitro, ex-vivo*, and *in vivo* models have been utilized for PK/PD modeling and each has advantages and disadvantages. Huang has established an *in vitro* dynamic model and studied the PK/PD relationship of tilmicosin against *M. gallisepticum*. The PK was simulated by the peristaltic pump and the PD was recorded by the change of *M. gallisepticum* number in the central compartment (12). The *in vitro* model was convenient to operate and to be cost-effective. It can be used to study the development of resistance between drug and bacterial interactions. However, it doesn't reflect the real situation, for the reason that the PK was simulated by the peristaltic pump and the immune defense of animals was ignored. In the current study, an *in vivo M. gallisepticum* infection model was established to evaluate the PK and PD of tilmicosin against *M. gallisepticum*-infected chickens. Although the *in vivo* model was time-consuming and expensive, the results were provided actually as close as possible.

The PK–PD integration parameters are essential for the formulation of optimal dosage regimens. Previous studies have indicated that the most suitable PK/PD parameter for macrolides with a long elimination half-life was related to AUC/MIC. For example, the PK/PD relationship of tildipirosin against *P. multocida* in a murine lung infection model indicated that AUC/MIC was the most appropriate PK/PD index (20). Gamithromycin possesses a rapid and concentration-dependent killing against *Haemophilus parasuis* and the AUC/MIC ratio correlated well in an *ex vivo* model (*R*^2^ = 0.97) (21). In the previous reports, Huang studied the PK/PD integration of tilmicosin against *M. gallisepticum* and *Mycoplasma hyopneumoniae* in the *in vitro* dynamic model. These studies also demonstrated that AUC/MIC was the best PK/PD parameter (*R*^2^ was 0.87 and 0.99, respectively) to predict the antimicrobial activity of tilmicosin against *M. gallisepticum* and *M. hyopneumoniae* (12, 13). Consequently, AUC/MIC was the preferable PK/PD index to investigate the PK/PD relationship of tilmicosin against *M. gallisepticum in vivo*.

Antibacterial resistance in *M. gallisepticum* has been increasing and is primarily the result of irrational antibiotic use and abuse (22, 23). To solve these types of problems, Zhao and Drlica proposed the “mutant selection window” (MSW) hypothesis that a drug concentration zone exists in which resistant mutants are selectively amplified and this generates reduced drug susceptibility (24). The MSW hypothesis has been verified *in vitro* and *in vivo* (25–27). The MSW of tilmicosin for *M. gallisepticum* strain S6 was determined *in vitro* and the MIC_99_ and MPC were determined to be 0.027 and 0.15 μg/ml, which corresponding to the lower and upper boundary of the MSW. The PK of tilmicosin in *M. gallisepticum-*infected chickens revealed that lung tilmicosin levels 24 h were partly within the MSW following oral single doses of 1, 2, 4, and 7.5 mg/kg and resistant mutant strains could possibly exist. These findings suggested that tilmicosin may be therapeutically effective to treat *M. gallisepticum* infection and restrict the acquisition of resistance in chickens if administered at a dosage >7.5 mg/kg after a single oral dose.

An additional study using *M. gallisepticum* infection of broiler chickens treated with tilmicosin at 10 and 20 mg/kg in the drinking water for five successive days found that respiratory tract lesions with 20 mg/kg treatment were significantly fewer than at 10 mg/kg (28). Consequently, a dosing scheme of 20 mg/kg of tilmicosin for five successive days was a logical conclusion for the dosing strategy to treat clinical outbreaks of *M. gallisepticum* in broilers. Our results indicated that 9.12 mg/kg tilmicosin after an oral single dose was sufficient for *M. gallisepticum* infection treatment. The different intracorporal processes of tilmicosin in chicken result from the different dosing regimens, such as the route, dose, and interval of the administration. These data provide a reliable basis for designing a rational treatment regimen for *M. gallisepticum* infections and the actual effect should be verified in clinical practice.

## Conclusion

The *in vivo M. gallisepticum* infection model was established and tilmicosin and the CFU of *M gallisepticum* in the lungs were taken as endpoints to evaluate PK/PD interactions. The PK/PD parameter of AUC/MIC correlated well with anti-mycoplasmal efficacy (*R*^2^ = 0.92). The ratios of AUC/MIC for 1 log_10_ and 3 log_10_ CFU/lung reductions were 300.02 and 6,950.15 h, respectively. These results indicated that tilmicosin at a dose of 9.12 mg/kg would be therapeutically effective to treat *M. gallisepticum* infections in chickens.

## Data availability statement

The original contributions presented in the study are included in the article/Supplementary material, further inquiries can be directed to the corresponding author/s.

## Ethics statement

The animal experiments were approved by the Animal Research Committees of Foshan University Animal Ethics Committee (Approval number: 2019044).

## Author contributions

Methodology, software, validation, formal analysis, data curation, writing original draft preparation, writing review and editing, visualization, project administration, and funding acquisition: NZ. Investigation and resources: NZ, JL, XY, MZ, SY, HY, HD, and DZ. Supervision: YB. All authors contributed to the article and approved the submitted version.

## Funding

This study was supported financially by the Guangdong Basic and Applied Basic Research Foundation (No. 2020A1515010664) and the Guangdong Province University Technology Service Special Project for Rural Revitalization Key Fields (No. 2017KQNCX212).

## Conflict of interest

The authors declare that the research was conducted in the absence of any commercial or financial relationships that could be construed as a potential conflict of interest.

## Publisher's note

All claims expressed in this article are solely those of the authors and do not necessarily represent those of their affiliated organizations, or those of the publisher, the editors and the reviewers. Any product that may be evaluated in this article, or claim that may be made by its manufacturer, is not guaranteed or endorsed by the publisher.
