# Supplementary material for: Pharmacokinetic and Pharmacodynamic integration of tilmicosin against Mycoplasma gallisepticum in the target infection site in chickens
Source: Front Vet Sci. 2022 Sep 29;9:952599. doi: 10.3389/fvets.2022.952599 (PMC9557078; doi:10.3389/fvets.2022.952599)
Supplement: Supplementary file 1 [file Data_Sheet_1.docx]

Supplementary data

**Table 1.** The concentration of tilmicosin in the lung tissues of chickens in the infected group after 1 mg/kg orally administration (μg/g)

| Time（h） | Chickens | | | | | | | |
| --- | --- | --- | --- | --- | --- | --- | --- | --- |
|  | 1 | 2 | 3 | 4 | 5 | 6 | 7 | 8 |
| 0.083 | ND | ND | ND | ND | ND | ND | ND | ND |
| 0.25 | ND | ND | ND | ND | ND | ND | ND | ND |
| 0.5 | ND | ND | ND | ND | ND | ND | ND | ND |
| 1 | ND | 0.02 | ND | ND | 0.02 | ND | ND | ___ |
| 2 | 0.05 | 0.01 | 0.10 | 0.09 | 0.03 | 0.08 | 0.02 | 0.02 |
| 4 | 0.05 | 0.09 | 0.10 | 0.10 | 0.02 | 0.10 | 0.10 | 0.06 |
| 6 | 0.09 | 0.08 | 0.05 | 0.07 | 0.19 | 0.06 | 0.15 | ___ |
| 8 | 0.24 | 0.12 | 0.03 | 0.03 | 0.04 | 0.12 | 0.03 | 0.20 |
| 12 | 0.19 | 0.05 | 0.16 | 0.08 | 0.15 | 0.09 | 0.15 | ___ |
| 24 | 0.11 | 0.11 | 0.08 | 0.11 | 0.11 | 0.11 | 0.12 | 0.07 |
| 48 | 0.05 | 0.05 | 0.11 | 0.06 | 0.06 | 0.04 | 0.08 | 0.08 |
| 72 | 0.07 | 0.04 | 0.03 | 0.03 | 0.07 | 0.06 | 0.04 | ___ |
| 96 | 0.02 | 0.02 | 0.02 | 0.03 | 0.03 | 0.04 | 0.03 | 0.04 |
| 120 | 0.01 | 0.02 | 0.01 | 0.05 | 0.02 | 0.03 | 0.01 | 0.02 |
| 144 | 0.02 | 0.02 | 0.02 | 0.03 | 0.03 | 0.01 | 0.01 | 0.03 |

Note：___：Sample missing；ND：no drug concentration detected

**Table 2.** The concentration of tilmicosin in the lung tissues of chickens in the infected group after 1 mg/kg orally administration (μg/g)

| Time（h） | Chickens | | | | | | | |
| --- | --- | --- | --- | --- | --- | --- | --- | --- |
|  | 1 | 2 | 3 | 4 | 5 | 6 | 7 | 8 |
| 0.083 | 0.08 | 0.11 | 0.07 | 0.08 | 0.08 | 0.10 | 0.18 | 0.08 |
| 0.25 | 0.38 | 0.24 | 0.07 | 0.31 | 0.39 | 0.21 | 0.20 | 0.09 |
| 0.5 | 0.42 | 0.40 | 0.47 | 0.07 | 0.15 | 0.09 | 0.41 | ___ |
| 1 | 0.24 | 2.17 | 0.54 | 0.39 | 0.45 | 0.29 | 0.33 | 0.41 |
| 2 | 2.00 | 1.68 | 1.55 | 2.17 | 1.76 | 1.32 | 3.39 | 1.18 |
| 4 | 1.76 | 2.37 | 4.64 | 0.91 | 2.22 | 2.73 | 1.13 | 2.82 |
| 6 | 2.90 | 6.60 | 2.51 | 2.36 | 4.24 | 6.46 | 4.70 | 4.94 |
| 8 | 7.31 | 4.77 | 6.70 | 4.01 | 8.26 | 4.53 | 5.18 | 4.16 |
| 12 | 8.76 | 3.58 | 3.16 | 4.76 | 6.03 | 8.54 | 10.12 | 4.54 |
| 24 | 5.32 | 5.14 | 4.12 | 3.78 | 5.03 | 5.84 | 6.02 | 5.94 |
| 48 | 3.39 | 3.49 | 4.28 | 1.19 | 2.22 | 5.17 | 2.90 | 2.25 |
| 72 | 2.91 | 2.30 | 2.65 | 2.78 | 0.79 | 2.02 | 2.82 | 3.18 |
| 96 | 1.12 | 1.44 | 0.94 | 2.97 | 1.64 | 2.22 | 0.97 | 0.74 |
| 120 | 1.00 | 0.95 | 1.55 | 0.88 | 2.76 | 0.92 | 1.57 | 1.09 |
| 144 | 0.71 | 0.75 | 1.22 | 0.35 | 1.25 | 1.45 | 0.83 | 0.97 |

Note：___：Sample missing；ND：no drug concentration detected


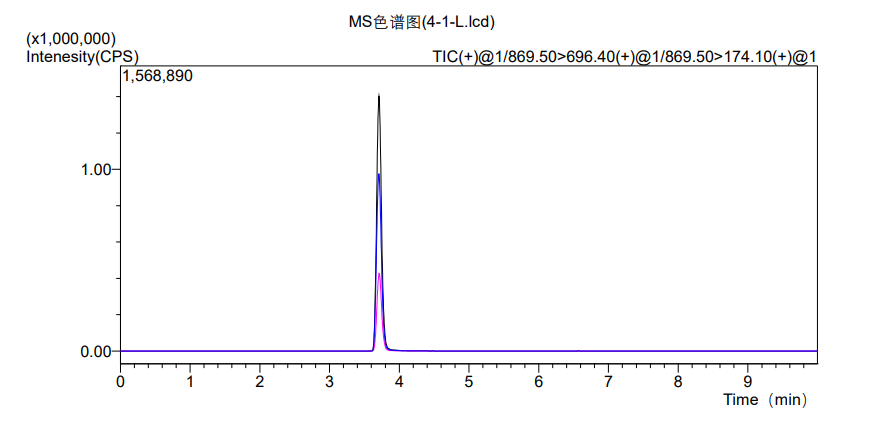


Figure 1. MRM chromatogram of tilmicosins: 0.02 μg/mL standard solution of tilmicosin ,
